# Ocular surface tumors

**DOI:** 10.4103/0974-620X.48415

**Published:** 2009

**Authors:** Ihab Saad Othman

**Affiliations:** Kasr El-Ainie Medical, School, Cairo University, Cairo, Egypt

**Keywords:** Conjunctival lymphoma, conjunctival melanoma, conjunctival nevi, conjunctvial neoplasms, corneal neoplasms, MALT, ocular surface tumors, PAM, topical chemotherapy

## Abstract

Tumors of the conjunctiva and cornea comprise a large and varied spectrum of conditions. These tumors are grouped into two major categories of congenital and acquired lesions. The acquired lesions are further subdivided based on origin of the mass into surface epithelial, mucoepidermoid, melanocytic, vascular, fibrous, neural, histiocytic, myxoid, myogenic, lipomatous, lymphoid, leukemic, metastatic and secondary tumors. Ocular surface tumors include a variety of neoplasms originating from squamous epithelium, melanocytic tumors and lymphocytic resident cells of the conjunctival stroma. In this review, we highlight clinical features of these lesions, important diagnostic and investigative tools and standard care of management.

## Method of Literature Search

The literature was searched on the Medline database, using the Pubmed interface including literature from the years 1984-2008. The search strategy included MeSH and natural language terms to retrieve references on conjunctival epithelial tumors, dysplasia, papilloma, conjunctival carcinoma in situ, squamous cell carcinoma, conjunctival melanosis, nevi, melanoma, and conjunctival lymphoma, including MALT lymphoma. Due to the fact that many large clinical controlled trials were not available, small patient series and relevant case reports were also included. For foreign language publications, no translation was obtained; however, the abstracts of non-English articles found to be of particular relevance to the review were utilized. Reference lists in retrieved articles, and textbooks, were also searched for relevant references.

## Introduction

The conjunctiva is a mucous membrane with a surface composed of nonkeratinizing stratified squamous epithelium intermixed with goblet and Langerhans cells.[1] Beneath conjunctival epithelium lies the conjunctival substantia propria, which is composed of a thin layer of loose connective tissue. Lymphocyte cell responses and antibody secretion by plasma cells in the conjunctival substantia propria are part of the eye’s normal defense mechanism.[1]

Tumors of the conjunctiva and cornea comprise a large and varied spectrum of conditions. These tumors are grouped into two major categories of congenital and acquired lesions. The acquired lesions are further subdivided based on origin of the mass into surface epithelial, melanocytic, vascular, fibrous, neural, histiocytic, myxoid, myogenic, lipomatous, lymphoid, leukemic, metastatic and secondary tumors.[2]

Ocular surface tumors include a variety of neoplasms originating from squamous epithelium, melanocytes, and lymphocytic resident cells of the conjunctival stroma. Ocular surface squamous neoplasia (OSSN) denotes neoplastic lesions of epithelial origin on the cornea and conjunctiva[3] and includes both squamous epithelial dysplasia and squamous cell carcinoma.

Melanocytic tumors include conjunctival nevi, melanosis (racial melanosis and primary acquired melanosis), melanoma and other ocular surface conditions like ocular melanocytosis and secondary pigmentary deposition. Lymphoid tumors of the conjunctiva include a variety of monoclonal and polycolonal lymphoid tumors.

In this report, we review ocular surface tumors diagnosis and management including squamous, melanocytic and lymphomatous lesions. Relevant literature search used the Medline database, using the Pubmed interface including literature from the years 1984-2008. Special emphasis was put on clinical relevance of information collected.

## Epidemiology and Pathogenesis

### 

#### Ocular surface squamous neoplasia

Ocular surface squamous neoplasia (OSSN) was first described by Lee and Hirst[3] as an umbrella term that encompasses intraepithelial and invasive squamous cell carcinoma of the conjunctiva and cornea. The incidence of OSSN ranges from 0.02 to 3.5 per 100 000 population and varies geographically with greater frequency near the equator. Generally, it is a slow growing tumor that rarely metastasises, but is capable of causing extensive local tissue destruction. Previously cited risk factors include advanced age, light skin pigmentation,[4] ultraviolet light B exposure,[5] tobacco smoke[6] and occupational exposure to petroleum products.[4,5] A role for human papillomavirus infection in the pathogenesis of OSSN has been suggested based on polymerase chain reaction evidence for human papillomavirus types 16 and 18 in OSSN lesions.[7] However, the reports relating to the association of HPV with conjunctival neoplasias are variable. This is attributed both to diversity of populations studied and/or absence of a “gold standard” test for HPV detection.[8]

Infection with human immunodeficiency virus (HIV) has also been associated with elevated rates of OSSN as it may be seen in 4% to 7.8% of persons with HIV,[9] particularly in sub-Saharan Africa.[10]

Xeroderma pigmentosa is an autosomal recessive inherited defect in DNA synthesis following inactivation by ultraviolet rays. It is associated with increased incidence of ocular surface neoplasia, and melanomas.

#### Clinical spectrum

The clinical spectrum of OSSN includes benign lesions like squamous papilloma, precancerous lesions as actinic keratosis, carcinoma in situ and invasive squamous carcinoma.

Squamous papilloma is a benign sessile or pedunculated proliferation of conjunctival epithelium seen as single or multiple cauliflower-like masses with central fibrovascular cores.[11] Many squamous papilloma are viral lesions (human papovavirus types 16, 18 and human papilloma virus) as detected by DNA hybridization.[12] Papillomata in children are multiple and recurrent. Table 1 shows main differences between papillomata affecting children and adults. Papilloma can show marked hypercellularity with cellular atypia, nuclear hyperchromatism and atypical mitoses (seen in 6%-27 % of cases) to be differentiated from carcinoma in situ versus squamous cell carcinoma.[11] Carcinoma rarely develops from conjunctival papilloma.

**Table 1:** Differences between pedunculated and sessile conjunctival papilloma

	*Pedunculated conjunctival papilloma*	*Sessile conjunctival papilloma*
Age	Children	Adults
Etiology	HPV-6, 11	HPV-16, 18
Site	Inferior fornix, semilunar fold	Limbus, may spread on cornea
Shape	Multiple branching fronds originating from a narrow base	Regular spaced fibrovascular fronds originating from a broad base
Recurrence	+++	+/-
Dysplasia	+/-	+++
Invasive carcinoma	-	+

#### Management of conjunctival papilloma

Even though conjunctival papilloma may persist for extended periods of time, with reported recurrence rates of 6%-27%,[11] the clinical course favors spontaneous regression and cure. In cases where large lesions cause symptoms or cosmetic defects, surgery remains the treatment of choice with double freeze-thaw cryotherapy to the remaining conjunctiva to prevent tumor recurrence.[13] Additionally, topical interferon alfa-2b[14] and mitomycin C[15] have been employed in the treatment of recurrent conjunctival papillomas. Immunomodulatory agents such as oral cimetidine have led to conjunctival papilloma regression.[16]

#### Conjunctival intraepithelial neoplasia (CIN, conjunctival dysplasia)

This includes a disease spectrum characterized by a replacement of the conjunctival epithelium by atypical squamous cells.

Clinically, CIN is an ill-defined gelatinous lesion that blends with surrounding normal conjunctiva. The surface mostly shows keratinization (leukoplakia) and the lesion is usually sessile (papilliform). The lesion shows abrupt transition between normal and acanthotic dysplastic epithelium, which may involve less than 50% of epithelial layer in mild dysplasia and more than 50% of thickness in severe dysplasia. The lesion may show a recurrence rate of up to 50% at 10 years with positive surgical margins.[1,2,17]

#### Carcinoma in situ(CIS)

**Figure 1:**
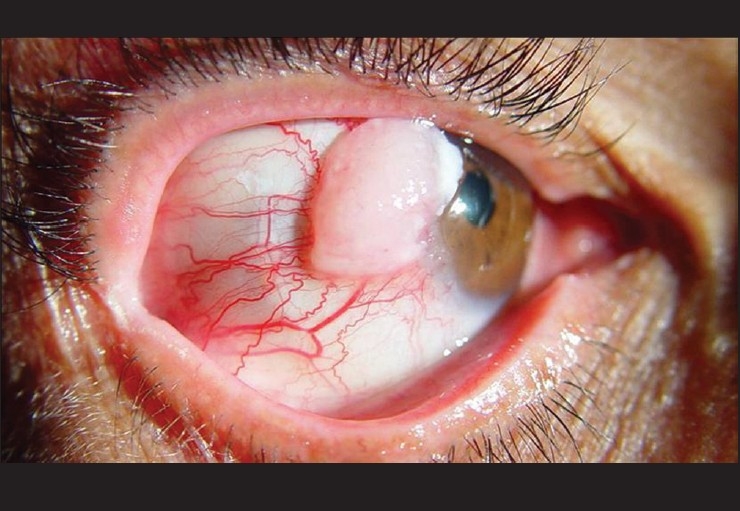
Photomicropgraph of ocular surface showing an ill-defined gelatinous mass encroaching on the cornea with feeder vessels. Surgical excision revealed conjunctival carcinoma *in situ*

It is a full thickness replacement of epithelium by frankly malignant cells: Epithelial basement membrane is intact, with no invasion into substantia propria. Clinically, it presents as an opalescent papillary masses at limbus with minimal leukoplakia [Figure 1]. Cells show loss of polarity and anaplasia. Spindle and epidermoid variants are detected on histopathology.[1,2,17]

#### Invasive squamous cell carcinoma (SCC)

SCC represents the final stage in malignancy evolution where malignant cells have broken through epithelial basement membrane invading substantia propria.

Clinically, it manifests as chronic irritation, with red eye, a raised gelatinous mass, and leukoplakia. Papillary masses appear at limbus with feeder vessels supplying the masses. Masses are initially mobile; the conjunctiva in later stages becoming fixed to the globe with deeper scleral infiltration. Two growth patterns are identified:[1,2]

- Exophytic or papillary growth pattern with growth in interpalpebral fissure
- Endophytic growth pattern with invasion of cornea, sclera, interior of globe, and posteriorly to the orbit

On microscopic examination, malignant epithelial cells invade the substantia propria and stroma. Spindle cell and mucoepidermoid variants represent a more aggressive tumor with early invasion and frequent recurrences.[18]

**Figure 2:**
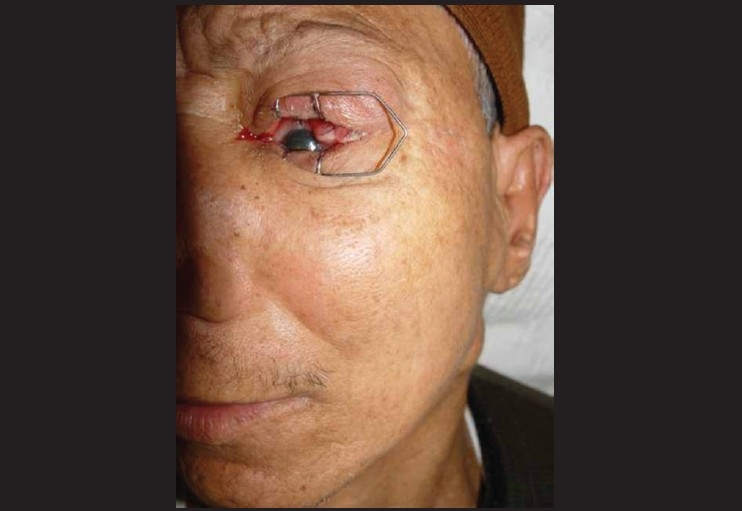
Photomicrograph showing conjunctival squamous cell carcinoma OS with preauricular and upper deep cervical lymph node metastasis. It is important to assess draining lymph nodes as art of exmaination of ocular surface tumors

Invasive squamous cell carcinoma may spread locally to the orbit, deeply to the corneal stroma, intraocular structures and sclera. Intraocular invasion has been reported in 2%-15% of all cases[19] and studies have found orbital invasion in 12%-16% of cases.[20] Tumor may rarely spread to the preauricular, submandibular and upper deep cervical lymph nodes [Figure 2]. Systemic spread is rare.

It may not be possible to distinguish dysplasia, Carcinoma in situ(CIS), and SCC on clinical grounds alone. In Lee and Hirst’s series of 288 patients with OSSN including 62 cases of SCC,[21] the overall clinical accuracy was 33% and only 30% for the SCC group. Macroscopic patterns of SCC have been described as gelatinous, velvety or papilliform, leucoplakic,[20–23] nodular and diffuse.[18]

#### Melanocytic ocular surface tumors

These include the following:

Benign melanocytic lesions:

- Benign epithelial melanosis
- Ocular melanocytosis
- Conjunctival Nevi

Preinvasive melanocytic tumors:

- Primary acquired melanosis

Malignant melanocytic tumors:

- Conjunctival melanoma

Benign epithelial melanosis is a flat, uninflamed, nonvascularized and finely to coarsely granular brown pigmentation generally occurring in the interpalpebral zone of the conjunctiva. The condition may be congenital or acquired as it is seen in dark skin persons with racial melanosis or climactic or chronic conjunctival irritation. Other predisposing factors include irradiation, arsenic poisoning, Addison’s disease, and chloasma of pregnancy. The lesion is usually bilateral and may be asymmetrical.[24]

Clinically, it is mostly located in the perilimbal and interpalpebral bulbar conjunctiva. The pigmentation moves with the conjunctiva. Microscopically, heavy pigmentation restricted to the basal cell layer of conjunctival epithelium, and the clear melanocytic cells are difficult to demonstrate with no nest formation. Minimal inflammation of substantia propria with normal lymphocytic population and scattered histiocytic melanophages are noted.[24] Squamous tumors in Blacks may be pigmented due to secondary acquired melanosis

Conjunctival Nevi are nests of benign nevus cells along epithelial base and/or within substantia propria. It is usually a congenital lesion which manifests during first 2 decades of life. It typically enlarges or become more pigmented at puberty or during pregnancy and may be amelanotic in 35% of cases with variable melanin contents.[25]

Clinically, it manifests as an elevated mass occupying the interpalpebral area where it is flat at the limbus and elevated at the bulbar conjunctiva and caruncle. It is usually pigmented in 65% of cases and may be amelanotic. As the mass is located in substantia propria, it is mobile with conjunctival movement. It shows intralesional epithelial inclusion cysts.[26]

Variants: According to the site of cell nesting and grouping, conjunctival nevi are classified as junctional nevi where nevus cells are confined to epithelial-subepithelial junction, compound nevi where nevus cells rest in both epithelium and subepitelium, and subepitehlial nevi where cells are entirely in substantia propria. Nevus cells presenting at epithelial-subepithelial junction in junctional and compound nevi may show malignant potential.[27]

#### Nevus of Ota (congenital oculodermal melanocytosis)

This is congenital melanosis of episclera with cutaneous involvement. The disease involves 1/2500 of the population and is more common in Black and Asian races.

Clinically, it appears as congenital slate gray periocular flat cutaneous pigmentation with an associated episcleral, orbital and uveal pigmentation. Episcleral pigmentation does not move with movement of conjunctiva

Histologically, it represents a superficial focal proliferation of subepithelial melanocytes with no cytological atypia.[28] From a prognostic point of view, it is important to examine and follow up the fundus of these patients with nevus of Ota, as the incidence of uveal melanoma is 0.4% (1/400 cases).[29] Also, these patients have a slightly more risk to develop orbital[30] and meningeal melanoma.

#### Primary acquired melanosis (PAM, Reese’s cancerous melanosis)

This is an acquired proliferation of melanocytes situated within the epithelium of the conjunctiva, commonly seen unilaterally in middle-age and elderly White population.[31] It presents clinically as multiple or single flat brown patches of superficial conjunctiva (rarely amelanotic) that move with movement of the conjunctiva. PAM has an insidious onset, and waxes and wanes. In 20% of cases, malignant melanoma develops from PAM and manifests clinically as an increase in vascularity and nodule formation.[26]

PAM can be differentiated histologically into two types:[24]

PAM without atypia:

- Epithelial hyperpigmentation with melanocytic hyperplasia
- Restricted to basilar region of epithelium without nuclear hyperchromatism or prominent nucleoli
- Low risk for conjunctival melanoma

PAM with atypia:

- Atypical melanocytic hyperplasia or malignant melanomain situinvolving conjunctival epithelium
- High risk for developing conjunctival melanoma:[32]
  -75% if PAM contains epithelioid cells
  -90% if intraepithelial pagetoid spread is present
  -20% if atypical melanocytes confined to basilar part of the epithelium
- Atypical cells confined to epithelium constitute radial growth phase
- Vertical growth phase: Represent invasive malignant melanoma

Management: Observe carefully with photographic documentation. Multiple map biopsies are warranted to detect atypia. Excision, cryotherapy and local chemotherapy are the different techniques to manage possible malignant transformation into melanoma.

#### Malignant melanoma of the conjunctiva

It is a relatively uncommon malignant conjunctival tumor arising from conjunctival melanocytes. The incidence is 1 in 2 millions in Fair complexion population with predisposing lesions primary acquired melanosis (75% of cases), preexisting conjunctival nevus (junctional or compound) (25%) or it may arise de novo (nodular melanoma).[33]

**Figure 3:**
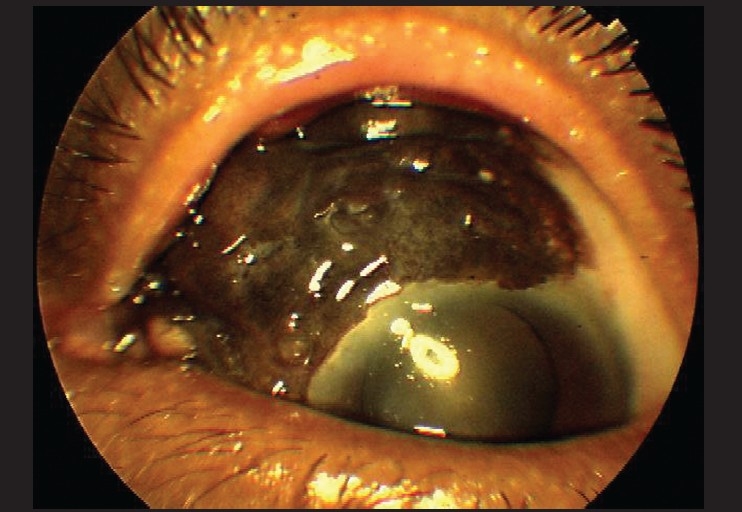
Photomicrograph showing diffuse conjunctival melanoma encroaching on the cornea OS. Melanoma arising from primary acquired melanosis with atypia is shown

Clinically, conjunctival melanoma arises from anywhere including bulbar, limbal, palpebral conjunctiva or the caruncle. It is commonly a nodular mass with variable pigmentation and shows feeder vessels with high intrinsic vascularity that leads to easy bleeding surface [Figure 3]. The tumor has the ability to locally spread and invades the globe and orbit and may show lymphatic spread to draining lymph nodes. Risk factors for metastasis include the following:[27]

- Younger age group
- Large tumor size
- Tumor extension to surgical margin
- Extralimbal location of the tumor
- Multicentricity
- Epithelioid cell type
- Lymphatic invasion
- Overall, metastatic disease from conjunctival melanoma has been found in 14% to 27% of patients, and 10 years after diagnosis, about 30% of patients have died of metastases.[27]
- Conjunctival melanoma has an unpredictable behavior with a mortality rate of 26 % at 10 years. Risk factors for death were found to be signs and symptoms of a lump, likely reflecting large tumor size and lack of associated primary acquired melanosis with the melanoma on pathologic examination. Large or thicker melanoma, whether arising in the skin or conjunctiva, carries a worse prognosis.[27]

Table 2 shows the main clinical differences between pigmented epibulbar and conjunctival masses

**Table 2:** Differential diagnosis of pigmented epibulbar lesions

	*Congenital melanosis*	*Primary acquired melanosis*	*Nevus*	*Malignant melanoma*
Location	Episcleral	Intraepithelial	Intraepithelial	Subepithelial
Movement with conjunctiva	No	Yes	Yes	Variable
Pigmentation	Slate gray	Variable	Variable	Variable
Special Features	Heterochromia iridis	Pseudocysts	Cystic contents	Feeder vessels
Course	Stationary	Waxes and wanes	Stationary	Progressive
Inflammation	-	+	+	++

#### Lymphoid lesions

The conjunctiva has a natural, submucosal reservoir of lymphoid tissue, the so-called conjunctiva-associated lymphoid tissue (CALT), which serves as a functionally active mucosal immune system.[34] Conjunctiva-associated lymphoid tissue may develop characteristics that are similar to acquired mucosa-associated lymphoid tissue (MALT) elsewhere in the body.[35] Neoplastic transformation of MALT leads to development of an extranodal (outside lymph nodes and spleen) marginal zone B-cell lymphoma of mucosa-associated lymphoid tissue, a MALT lymphoma. The term *MALT lymphoma* denotes a characteristic arrangement of lymphoid tissue found in certain mucosal surfaces, having distinct features from other forms of primary non-Hodgkin extranodal lymphoma. Mucosa-associated lymphoid tissue lymphoma constitutes more than two-thirds of ocular adnexal lymphoma[36] and in one investigation, 83% of patients with a conjunctival lymphoma had a conjunctival MALT lymphoma (CALT lymphoma).[37]

Mucosa-associated lymphoid tissue lymphoma develops as a result of prolonged antigen stimulation, leading to loss of regulation of B-lymphocyte proliferation and differentiation.[38] Thus, gastric MALT lymphoma has been found to develop as a result of chronic antigen stimulation caused by *Helicobacter pyloriinfection*.[39–41] The role of *H. pyloriin* development of conjunctival and ocular adnexal tumors remains controversial.[42,43]

Recently, a study of 40 patients with ocular adnexal lymphoma provided evidence for an association of *Chlamydia psittaci* and ocular adnexal lymphomas.[44] *C. psittaci* was found in 32 of 40 patients, and subsequent antibiotic treatment of 4 patients with doxycycline resulted in disease regression in 2 patients.[44] However, several infectious agents seem to take part in the pathogenesis of CALT lymphoma.*Chlamydia pneumoniae* has been identified in a patient with orbital MALT lymphoma,[45] and in another study,*H. pylori* DNA was detected in 4 out of 5 cases of conjunctival MALT lymphoma.[46] It is well known that B lymphocytes undergo malignant transformation resulting from the persistent antigen stimulus.[39] B lymphocytes in the inflammatory response of conjunctivitis may undergo a similar malignant transformation resulting from a chronic infection. Candidate pathogens are probably to be found among the group of infectious or allergic agents that are known to cause chronic conjunctivitis.

**Figure 4:**
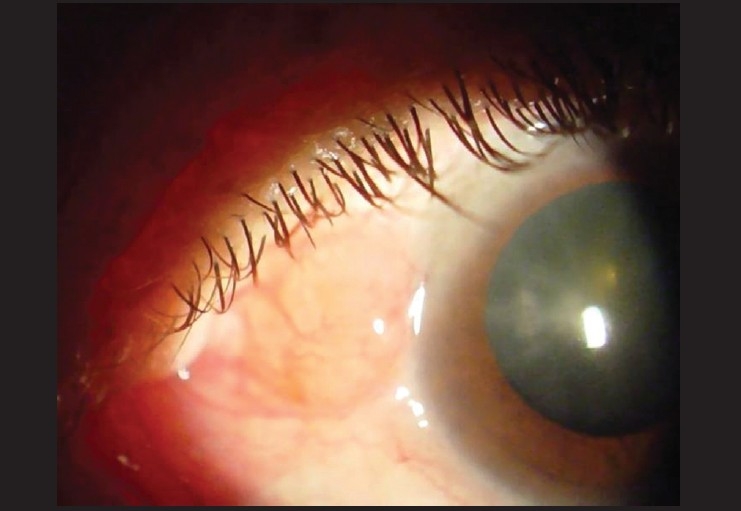
Photomicrograph showing subconjunctival Salmon-colored mass diagnostic of conjunctival lymphoma

Primary lymphomas of the conjunctiva are rare. Many of those in the conjunctiva are a discrete clinicopathologic entity of the marginal zone B-cell lymphoma of the MALT type, a low-grade non-Hodgkin B cell tumor with good prognosis.[1] Clinically, conjunctival MALT lymphomas may cause few symptoms because the neoplastic lymphocyte population lacks a connective tissue stroma and is able to mold to surrounding tissue without causing much irritation.[17] The disease follows an indolent course and mostly remains localized to the conjunctiva. Rarely, associated intraocular involvement has been described.[47] The vascularity of tumors is reflected by the characteristic “salmon patch” or fish-flesh appearance and are readily movable over epibulbar surface [Figure 4]. In a large case series, Shields *et al*, documented that conjunctival lymphoid tumors are generally hidden under the eyelid in the midbulbar conjunctiva or in the fornix, rather than at the more obvious limbal location, and hence, eyelid elevation and ocular rotation at the slitlamp are instrumental in tumor visualization.[48] Biopsy is necessary to establish the diagnosis as it is clinically difficult to differentiate between a benign inflammation and malignant lymphoid tumor, and a systemic evaluation should be performed in all affected patients to exclude the presence of systemic lymphoma.[13]

While isolated conjunctival MALT lymphoma is most often treated with external beam irradiation,[49] care must be taken to avoid complications such as xerophthalmia, keratitis, cataract formation, and retinal damage. A few case series have demonstrated that careful observation following biopsy may result in no inferior clinical outcomes or decreased survival.[50] Cryotherapy,[51] topical mitomycin C chemotherapy,[52] local interferon alpha[53] and rituximab[54] have been shown to be as effective as radiation with lower side-effects, although in smaller case series. Recently, consistent with its effectiveness in other non-Hodgkin B-cell lymphomas, at least one study has documented the efficacy of anti-CD20 monoclonal antibody therapy in causing tumor remission in two cases of relapsed conjunctival MALT lymphoma.[55]

Finally, recent studies suggest *Chlamydia psittaci* as an etiologic factor for conjunctival MALT lymphoma, and showed regression of ocular adnexal lymphoma after *C. psittaci* -eradicating antibiotic therapy.[56,57] However, more evidence has to be present showing the causal relationship of chronic infection by *C. psittaci* or other agents before generalizing the use of antibiotics in management of adnexal lymphomas.

## Diagnosis and Investigations of Ocular Surface Tumors

When faced with an ocular surface tumor, several points have to be resolved in order to reach a proper anatomical, clinical and pathological diagnosis as well as extent of the tumor and its complications:

- Site, size, shape, surface, feeder vessels, consistency, and exact anatomical location of the lesion whether it is conjunctival (moves with conjunctiva when applying topical anesthesia with a cotton tipped applicator), subconjunctival or episcleral where the conjunctiva glides over the surface of the lesion (PAM, nevus of Ota) or is it fixed to the globe (invading squamous cell carcinoma).
- Assessment of the extent of the lesion:
  - Intraocular involvement: It is important in lesions encroaching on the cornea and involving the limbus to assess intraocular angle involvement with gonioscopy and assess the degree of angle involvement. Ultrasound biomicroscopy offers an accurate tool to delineated scleral and/or intraocular involvement of the lesion. MRI scans focusing on the anterior orbit, with Gadolinium enhancement using 2 mm cuts may add additional information to the degree of eyeball involvement.
  - Assess orbital extension: Clinically, a superficial malignant mass (SCC or conjunctiva MM) may extend beyond the fornices to the anterior orbit. By using CT scans and MRI scans the degree and accurate extent of the lesions can be identified.
  - Lymph node spread: It is a crucial part of the clinical examination to assess preauricular, submandibular and upper deep cervical lymph nodes. A trained ophthalmologist should ask a veiled lady to unveil to examine these important structures to assess the degree of lymph nodes involvement.
- Pathological diagnosis:
  - After initial examination, one should reach a possible diagnosis or a differential diagnosis whether this is a squamous lesion (leukoplakia), melanotic lesion, lymphomatous (salmon patch) or others. Further investigations help confirm the clinical differential diagnosis.
  - Impression cytology (IC): The development of diagnostic techniques such as impression cytology is of value in clinical decision making and follow-up management.
  - c. Recently, the use ofin vivoconfocal microscopy could assist as a noninvasive tool in differential diagnosis and follow-up of pigmented conjunctival tumors.[62]

### 

#### Role of IC in OSSN

- Mapping of conjunctival surface in multifocal disease: impression cytology studies indicate the likelihood that ocular surface dysplasia is a multifocal disease, with clinically normal areas of the limbus being positive for dysplasia at areas remote from the clinical tumor.
- Impression cytology in ocular surface neoplasia has high positive predictive accuracy of 97.4% compared with tissue histology. However, a fair negative predictive accuracy of 52.9% indicates that impression cytology is a valuable screening technique, but it is not a “gold standard”.[58]

#### Technique

Small cellulose acetate strips and Biopore membrane offer greater sampling flexibility. The use of the Papanicolaou stain is strongly recommended when examining cytological preparations for squamous neoplasia

#### Findings and interpretation

- Normal cytology: The surface layers of normal conjunctival epithelium, a stratified cuboidal to columnar epithelium with mucin secreting goblet cells, are usually readily removed by IC and are often seen as large cohesive sheets. The conjunctival epithelium merges into the nonkeratinized stratified squamous epithelium of the cornea at the limbus. Because healthy corneal cells are tightly bound to each other and to their basement membrane, only a few ragged intermediate squamous cells are released usually.[59]
- Squamous surface neoplasia: In contrast, neoplastic cells usually are readily obtained from both areas of the ocular surface. For intraepithelial neoplasia, keratinized dysplastic cells are often accompanied by hyperkeratosis, syncytial-like groupings, and nonkeratinized dysplastic cells. Within the invasive group, cases with significant keratinization or little keratinization and sometimes also prominent macronucleoli are described.[59]

#### Role of IC on ocular melanocytic lesions

Impression cytology using biopore membrane has an important role in diagnosing conjunctival melanoma or PAM with severe dysplasia as it shows superficial atypical melanocytes and their proportion relative to benign epithelial cells.[60] However, benign conditions such as conjunctival nevi or PAM without atypia, where nevus cells or atypical melanocytes do not ascend superficially through conjunctival epithelium, the positive predictive value of impression cytology is less evident.[61]

## Management of Precancerous and Cancerous Ocular Surface Tumors

### 

#### Introduction

The “gold standard” treatment in cases of ocular surface neoplasia (OSSN) and conjunctival melanoma rests mainly on the complete surgical eradication in the form of excisional biopsy, accompanied by resection of a wide tumor-free margin of 3-4 mm. Unfortunately, residual tumor cells left in the bordering tissues carry the risk of frustrating recurrences. A successful treatment depends principally on the absolute eradication of tumor cells from the ocular surface.[63,64] Adjuvant therapeutic measures, including cryotherapy,[65] alcohol epitheliectomy,[66,67] radiation[68,69] and/or other medical therapies, including mitomycin C and interferon are also used in the management.[43]

#### Surgery

When dealing with ocular surface premalignant or malignant conditions, it is important to standardize surgical technique to get a radical tumor excision with no residual malignant cells that would induce frustrating recurrences. The “no touch” technique popularized by Shields *et al*,[63] is widely accepted as a standard approach. It is also important to remember to keep the operative field dry and avoid squirting of BSS on the cornea (to keep it wet) as the jet of BSS might disseminate tumor cells in fornices.

#### Surgical technique[63]

**Figure 5:**
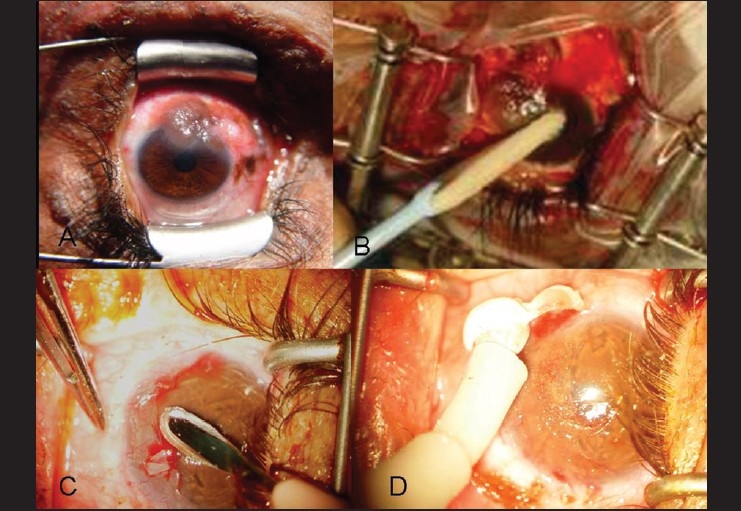
Principles of management: A: Photomicrograph showing conjunctival mealnoma. B: Alcohol epitheliectomy. C: Superficial keratectomy/Sclerectomy D: Cryotherapy with double freeze thaw technique. It is important to keep field dry

This technique includes removal of a 4-mm clinically tumor-free conjunctival skirt peripheral to the tumor and a thin scleral flap beneath the tumor when sclera is involved. The corneal epithelium 2 mm anterior to the tumor is treated with absolute alcohol and gently scraped toward the tumor edge, and the entire lesion sharply dissected off the corneal limbus. Cryotherapy of the cut conjunctival edge is performed with a double-freeze thaw technique, and the limbal and scleral base of the tumor treated with absolute (100%) alcohol. The conjunctival edge is secured to the sclera with episcleral 10-0 nylon sutures [Figure 5].

The surgeon may face a surgical dilemma in these challenging situations regarding the optimal technique for reconstructing the excised areas. In cases where extensive resections are performed, reconstruction may not be viable with either conjunctival free or rotational conjunctival grafts and/or mucosal autograft[70,71] or even lamellar keratoplasty. To avoid such extensive surface reconstruction, there may be a tendency to create relatively smaller defects, which in some instances may even contribute to the increase of recurrences. Furthermore, large tissue removal and the accompanying inflammation secondary to adjunctive cryotherapy and/or other conventional therapies might result in a myriad of fearsome collateral damages, among them partial limbal stem cell deficiency,[72] compromised wound healing, scarring, granulation tissue formation, motility restriction, and partial or total limbal stem cell deficiency,[73] corneal pannus and scarring, symblepharon, pseudopterygium,[74,75] cataract, uveitis, and retinal breaks.[66]

#### Use of amniotic membrane (AM) in ocular surface reconstruction

The amniotic membrane (AM) is the innermost layer of the placenta and consists of a thick basement membrane and avascular stroma. It is used as a patch or a graft to cover ocular surfaces after tumor excision and reconstruction. Its use is to provide epithelial cover, to reduce inflammation, to reduce vascularization, to limit scarring and to arrest melting.[76] Amniotic membrane has anti-adhesive properties, thus promoting epithelialization, and decreasing neovascularization, inflammation, and fibrosis. There are no histocompatibility issues with the recipient as amniotic membrane has no HLA antigens.[77]

AM offers an advantage over buccal or nasal mucosal autografts, which invariably result in a non-conjunctival epithelial morphology. The AM reconstructed conjunctival surface retains a normal conjunctival epithelial phenotype as shown by impression cytology.[78] AM can restore the corneal surface without being complicated with limbal stem cell deficiency.[77] This result is in agreement with the finding that AM alone without concomitant transplantation of autologous or allogeneic limbal epithelial stem cells is amenable to treat pre-existing partial limbal stem cell deficiency.[79] Lately, in vitro[80] and in vivo[81] studies have shown that AM serves as a suitable substrate for expanding ocular surface epithelia and subsequent transplantation. Many clinical reports[80,81] strongly support the fact that AM is a suitable tissue for the expansion and survival of epithelial cells.[82]

The lack of inflammation and scarring is particularly important when extensive resections are performed in conjunction with cryotherapy because the increased inflammation may even compromise the ocular motility or cause symblepharon. The final cosmetic appearance meets patients’ satisfaction in almost all cases.

#### Standard of care in management of ocular surface squamous neoplasia

The classical approach to the treatment of squamous neoplasias of the ocular surface is based on surgical resection and cryotherapy. High rates of recurrence have been demonstrated if the margins are not free after the resection. Clear surgical margins are notoriously difficult to obtain, most likely due to diffuse lateral or basal growth of the lesions and the inherent difficulty in interpreting frozen or permanent sections of conjunctival and corneal specimens.

Reported recurrence rates after surgical treatment are significant.[18,19,23] In the published study with the longest follow-up, 33% of patients with negative conjunctival margins at surgery and 56% with positive margins had experienced recurrence of their tumor by 10 years after excision.[83]

The standard of care for the treatment of ocular surface squamous neoplasia appears to have shifted from surgery toward the use of topical chemotherapeutic agents as adjuncts to surgery or even in some instances as sole therapy, despite a paucity of long-term studies in the published literature. This was initiated from observations of the effect of chemotherapeutic agents on inducing tumor regression or even disappearance without surgery, and their effects in cases with positive tumor margins.

Primary or adjuvant chemotherapy with mitomycin C (MMC) or 5-fluorouracil has been employed to treat these neoplasias, but severe side effects on the ocular surface have been described. Interferon (INF) alpha 2b is less toxic to the ocular surface. MMC is most widely used by external disease specialists.[84]

#### Mitomycin C

Mitomycin C is an alkylating antibiotic that acts in all phases of the cell cycle inducing scission of tumor DNA even after treatment has been discontinued, thereby mimicking the effects of ionizing radiation.[85] Rapidly dividing cells are most sensitive. The greatest chance of eradicating all tumor cells is achieved by using the highest possible doses against the smallest possible tumor load.[86] These properties of mitomycin C support its potential chemotherapeutic effectiveness as an adjunct to surgical excision.

#### Drug formulation

Two milligrams of mitomycin C is reconstituted with 5 ml of sterile water for injection in the clinical pharmacy. The preparation is performed using safe-handling and aseptic techniques using a 23-gauge needle and syringe fitted with a 0.22-µm filter. The solution is injected into a dark glass bottle through the dropper nozzle, making sure that there is no positive pressure in the bottle by first withdrawing air. The bottles are labeled and given a 14-day expiry date from preparation and 7 days after being opened.[87]

#### Drug administration

Topical mitomycin 0.4 mg/ml (0.04%) is administered 4 times a day for 3 weeks. Two separate bottles are provided at day 1 of the treatment trial, and the third bottle is provided at the end of 2 weeks of treatment; the patient is instructed to store these in the refrigerator and use 1 bottle per week of the treatment. Patients are instructed to avoid contact of the drop to the skin. Disposable gloves are advised to be used during handling drops. Cytotoxic marked plastic bags are given for storage of all materials potentially or actually contaminated with mitomycin, and these are returned to the investigation center for appropriate disposal.[87]

It is preferable that the patients would have upper and lower collagen punctal plugs inserted just before starting on the drugs. If not feasible, the patient is instructed to occlude the punctae and canalicular area manually and to keep his or her eyes closed for 2 min after the drop was instilled. These maneuvers are instituted to minimize contact of MMC to nasal mucosa and gastrointestinal (GI) absorption of the mitomycin.[87]

#### Mode of action

Mitomycin-C selectively inhibits DNA synthesis and is cell cycle-nonspecific. Consequently, the rapid cycling tumor cells are treated, while the slow cycling stem cells are probably left unaffected This results in scission of tumor DNA even after the treatment is discontinued.[86] This effect should inhibit both the neoplastic tissue and the fibrovascular response after cryotherapy (symblepharon formation). Secondly, topical mitocmycin C can treat satellite and multifocal lesions and the entire ocular surface, obviating the need to establish margins of excision.[88]

#### The role of MMC in conjunctival intraepithelial neoplasia

Mitomycin C was found to be useful as primary therapy for CIN.[87] It is also widely used as adjunct to surgery for extensive disease with positive surgical margins, multicentric tumor and recurrent tumor after surgical excision.[89–92]

#### The role MMC in invasive SCC

Topical mitomycin has been used successfully as adjunctive therapy in controlling conjunctival and corneal SCC even in extensive recurrent disease. It has been used solely in a concentration of 0.04%[93] and has been combined with topical cyclosporine 0.05% with a lesser concentration of MMC (0.01%).[94]

#### The role of MMC in melanocytic surface tumors

Topical MMC was found to be effective as a primary or adjuvant therapy in management of PAM with atypia.[95] MMC seems to be ineffective as a primary therapy in nodular melanoma as subepithelial tumor nests seem to be resistant to treatment.[96] MMC may have some role as adjuvant following surgery for extensive conjunctival melanoma or in severe limbal stem cell deficiency.[97]

Rates of tumor regression for CIN and squamous cell carcinoma ranged from 80% to 96%, and 70% of pigmented tumors regressed after an average follow-up of 27 months

#### Side effects and complications of MMC

Toxicity to topical mitomycin C is widely reported. Patients most frequently experience transient keratitis, redness, and irritation. Wilson *et al*[92] and Demirci *et al*,[95] described transient side effects like tearing, photophobia, and punctate epitheliopathy in all patients receiving 0.04% mitomycin C eye drops, but no serious complications were reported. Ando *et al*,[98] concluded that 0.04% mitomycin C was relatively nontoxic to intact corneal epithelium. Intermittent therapy prevents damage to slower growing cells, allowing them to repair their DNA, and limbal stem cell depletion can be avoided.[87,99]

#### Interferon alfa-2b

Interferons are a family of glycoprotein molecules that act at cell surface receptors to produce antiviral and antitumor activities. Topical IFNα2b is the recombinant form of interferon-a.[100]

#### Drug formulation

The topical IFNα2b drops (1 million IU/ml) are prepared via dilution of the injectable recombinant IFNα2b (Intron A [Schering Plough, Kenilworth, NJ], 6 million IU/ml) with preservative-free balanced salt solutions.[101]

#### Drug administration

Patients are instructed to refrigerate the preparation between uses, and they are treated with topical IFNα2b 4 times daily until lesion resolution is noted.[101]

#### Mode of action of interferon alfa-2b

A mechanism of action for interferon alfa-2b in OSSN is uncertain but may include inhibition of angiogenesis[102] and/or human papillomavirus replication.[103]

#### The role of interferon alfa-2b in conjunctival intraepithelial neoplasia

The absence of serious side effects after topical administration of INF alpha 2b leads to the recommendation of this modality of therapy for primary and recurrent cases of CIN. The use of subconjunctival or perilesional[104] injection and topical IFNa2b for primary, recalcitrant and recurrent corneal and conjunctival intraepithelial neoplasia has shown impressive rates of disease resolution over varying follow-up periods.[105–110]

#### Side effects and complications

Overall, reports in the literature demonstrate that topical IFNa2b has minimal side effects, without ocular surface toxicity or carcinogenic potential. Minor side effects of topical interferon alfa-2b have been reported, such as follicular conjunctivitis and conjunctival injection.[111] In contrast to topical mitomycin C, interferon alfa-2b exhibits no intrinsic toxicity when applied topically and has no known carcinogenic potential.[112]

#### Interferon alpha-2b in conjunctival melanoma

Interferon alfa-2b was used as adjuvant therapy in patients with recurrent conjunctival melanoma or as a substitute to MMC and was found efficient in inducing regression in corneal and conjunctival melanoma.[113]

#### Orbital exenteration

Secondary intraorbital spread of malignant ocular surface tumors to the fornices, eyelids, anterior orbit is the most common indication for exenteration. A lid-sparing exenteration is performed in cases of tumor sparing musculocutaneous layer of eyelids.[114]

## References

[1] Yanoff M, Fine BS, Yanoff M, Fine BS (2002). Conjunctiva. Ocular pathology.

[2] Shields CL, Shields JA (200). Conjunctival tumors in children. Curr Opin Ophthalmol.

[3] Lee GA, Hirst LW (1995). Ocular surface squamous neoplasia. Surv Ophthalmol.

[4] Kiire CA, Dhillon B (2006). The aetiology and associations of conjunctival intraepithelial neoplasia. Br J Ophthalmol.

[5] Lee GA, Williams G, Hirst LW, Green AC (1994). Risk factors in the development of ocular surface epithelial dysplasia. Ophthalmology.

[6] McDonnell JM, Mayr AJ, Martin WJ (1989). DNA of human papillomavirus type 16 in dysplastic and malignant lesions of the conjunctiva and cornea. N Engl J Med.

[7] Scott IU, Karp CL, Nuovo GJ (2002). Human papillomavirus 16 and 18 expression in conjunctival intraepithelial neoplasia. Ophthalmology.

[8] Eng HL, Lin TM, Chen SY, Wu SM, Chen WJ (2002). Failure to detect human papillomavirus DNA in malignant epithelial neoplasm of conjunctiva by Polymerase chain reaction. Am J Clin Pathol.

[9] Karp CL, Scott IU, Chang TS, Pflugfelder SC (1996). Conjunctival intraepithelial neoplasia: A possible marker for human immunodeficiency virus infection?. Arch Ophthalmol.

[10] Newton R, Ziegler J, Beral V, Mbidde E, Carpenter L, Wabinga H (2001). A case-control study of human immunodeficiency virus infection and cancer in adults and children residing in Kampala, Uganda. Int J Cancer.

[11] Sjö N, Heegaard S, Prause JU (2000). Conjunctival papilloma: A histopathologically based retrospective study. Acta Ophthalmol Scand.

[12] McDonnell JM, McDonnell PJ, Mounts P, Wu TC, Green WR (1986). Demonstration of papillomavirus capsid antigen in human conjunctival neoplasia. Arch Ophthalmol.

[13] Shields CL, Shields JA (2004). Tumors of the conjunctiva and cornea. Surv Ophthalmol.

[14] Schechter BA, Rand WJ, Velazquez GE, Williams WD, Starasoler L (2002). Treatment of conjunctival papillomata with topical interferon Alfa-2b. Am J Ophthalmol.

[15] Yuen HK, Yeung EF, Chan NR, Chi SC, Lam DS (2002). The use of postoperative topical mitomycin C in the treatment of recurrent conjunctival papilloma. Cornea.

[16] Shields CL, Lally MR, Singh AD, Shields JA, Nowinski T (1999). Oral cimetidine (Tagamet) for recalcitrant, diffuse conjunctival papillomatosis. Am J Ophthalmol.

[17] Eagle RC (1999). Eye pathology: An atlas and basic text.

[18] McKelvie PA, Daniell M, McNab A, Loughnan M, Santamaria JD (2002). Squamous cell carcinoma of the conjunctiva: A series of 26 cases. Br J Ophthalmol.

[19] Tunc M, Char DH, Crawford B, Miller T (1999). Intraepithelial and invasive squamous cell carcinoma of the conjunctiva: Analysis of 60 cases. Br J Ophthalmol.

[20] Erie JC, Campbell RJ, Liesegang TJ (1986). Conjunctival and corneal intraepithelial and invasive neoplasia. Ophthalmology.

[21] Lee GA, Hirst LW (1997). Retrospective study of ocular surface neoplasia. Aust NZ J Ophthalmol.

[22] Blodi FC (1973). Squamous cell carcinoma of the conjunctiva. Doc Ophthalmol.

[23] Sudesh S, Rapuano CJ, Cohen EJ, Eagle RC, Laibson PR (2000). Surgical management of ocular surface squamous neoplasms: The experience from a cornea center. Cornea.

[24] Jakobiec FA (1984). The ultrastructure of conjunctival melanocytic tumors. Trans Am Ophthalmol Soc.

[25] Folberg R, Jakobiec FA, Bernardino VB, Iwamoto T (1989). Benign conjunctival melanocytic lesions: Clinicopathologic features. Ophthalmology.

[26] Jakobiec FA, Folberg R, Iwamoto T (1989). Clinicopathologic characteristics of premalignant and malignant melanocytic lesions of the conjunctiva. Ophthalmology.

[27] Shields CL (2000). Conjunctival melanoma: Risk factors for recurrence, exenteration, metastasis and death in 150 consecutive patients. Tr Am Ophth Soc.

[28] Infante de German-Ribon R, Singh AD, Arevalo JF, Driebe W, Eskin T (1999). Choroidal melanoma with oculodermal melanocytosis in Hispanic patients. Am J Ophthalmol.

[29] Singh AD, De Potter P, Fijal BA, Shields CL, Shields JA, Elston RC (1998). Lifetime prevalence of uveal melanoma in white patients with oculo (dermal) melanocytosis. Ophthalmology.

[30] Wilkes TD, Uthman EO, Thornton CN, Cole RE (1984). Malignant melanoma of the orbit in a black patient with ocular melanocytosis. Arch Ophthalmol.

[31] Sugiura M, Colby KA, Mihm MC, Zembowicz A (2007). Low-risk and high-risk histologic features in conjunctival primary acquired melanosis with atypia: Clinicopathologic analysis of 29 cases. Am J Surg Pathol.

[32] Gloor P, Alexandrakis G (1995). Clinical characterization of primary acquired melanosis. Invest Ophthalmol.

[33] Missotten GS, Keijser S, De Keizer RJ, De Wolff-Rouendaal D (2005). Conjunctival melanoma in the Netherlands: A nationwide study. Invest Ophthalmol Vis Sci.

[34] Knop N, Knop E (2000). Conjunctiva-associated lymphoid tissue in the human eye. Invest Ophthalmol Vis Sci.

[35] Wotherspoon AC, Diss TC, Pan LX, Schmid C, Kerr-Muir MG, Lea SH (1993). Primary low-grade B-cell lymphoma of the conjunctiva: A mucosa-associated lymphoid tissue type lymphoma. Histopathology.

[36] Auw-Haedrich C, Coupland SE, Kapp A, Schmitt-Gräff A, Buchen R, Witschel H (2001). Long term outcome of ocular adnexal lymphoma subtyped according to the REAL classification. Br J Ophthalmol.

[37] Coupland SE, Krause L, Delecluse HJ, Anagnostopoulos I, Foss HD, Hummel M (1998). Lymphoproliferative lesions of the ocular adnexa: Analysis of 112 cases. Ophthalmology.

[38] de Jong D, Aleman BM, Taal BG, Boot H (1999). Controversies and consensus in the diagnosis, work-up and treatment of gastric lymphoma: An international survey. Ann Oncol.

[39] Du MQ, Isaccson PG (2002). Gastric MALT lymphoma: From aetiology to treatment. Lancet Oncol.

[40] Thiede C, Morgner A, Alpen B (1997). What role does Helicobacter pylori eradication play in gastric MALT and gastric MALT lymphoma?. Gastroenterology.

[41] Nobre-Leitao C, Lage P, Cravo M, Cabeçadas J, Chaves P, Alberto-Santos A (1998). Treatment of gastric MALT lymphoma by Helicobacter pylori eradication: A study controlled by endoscopic ultrasonography. Am J Gastroenterol.

[42] Isaacson PG, Müller-Hermelink HK, Piris MA, Jaffe ES, Harris NL, Stein H, Vardiman JW (2001). Extranodal marginal zone B-cell lymphoma (MALT lymphoma). Pathology and Genetics of Tumours of Haematopoietic and Lymphoid Tissues, World Health Organization Classification of Tumours.

[43] Verma V, Shen D, Sieving PC, Chan CC (2008). The role of infectious agents in the etiology of ocular adnexal neoplasia. Surv Ophthalmol.

[44] Ferreri AJ, Guidoboni M, Ponzoni M, De Conciliis C, Dell’Oro S, Fleischhauer K (2004). Evidence for an association between Chlamydia psittaci and ocular adnexal lymphomas. J Natl Cancer Inst.

[45] Shen D, Yuen HK, Galita DA, Chan NR, Chan CC (2006). Detection of Chlamydia pneumoniae in a bilateral orbital mucosa-associated lymphoid tissue lymphoma. Am J Ophthalmol.

[46] Chan CC, Smith JA, Shen DF (2004). Helicobacter pylori (H. pylori) molecular signature in conjunctival mucosa-associated lymphoid tissue (MALT) lymphoma. Histol Histopathol.

[47] Sarraf D, Jain A, Dubovy S, Kreiger A, Fong D, Paschal J (2005). Mucosa-associated lymphoid tissue lymphoma with intraocular involvement. Retina.

[48] Shields CL, Shields JA, Carvalho C, Rundle P, Smith AF (2001). Conjunctival lymphoid tumors: Clinical analysis of 117 cases and relationship to systemic lymphoma. Ophthalmology.

[49] Uno T, Isobe K, Shikama N, Nishikawa A, Oguchi M, Ueno N (2003). Radiotherapy for extranodal, marginal zone, B-cell lymphoma of mucosa-associated lymphoid tissue originating in the ocular adnexa: A multiinstitutional, retrospective review of 50 patients. Cancer.

[50] Tanimoto K, Kaneko A, Suzuki S, Sekiguchi N, Maruyama D, Kim SW (2006). Long-term follow-up results of no initial therapy for ocular adnexal MALT lymphoma. Ann Oncol.

[51] Eichler MD, Fraunfelder FT (1994). Cryotherapy for conjunctival lymphoid tumors. Am J Ophthalmol.

[52] Yu CS, Chiu SI, Ng CS, Chan HH, Tse RK (2008). Localized conjunctival mucosa-associated lymphoid tissue (MALT) lymphoma is amenable to local chemotherapy. Int Ophthalmol.

[53] Blasi MA, Gherlinzoni F, Calvisi G, Sasso P, Tani M, Cellini M (2001). Local chemotherapy with interferon-alpha for conjunctival mucosa-associated lymphoid tissue lymphoma: A preliminary report. Ophthalmology.

[54] Tsai PS, Colby KA (2005). Treatment of conjunctival lymphomas. Semin Ophthalmol.

[55] Nückel H, Meller D, Steuhl KP, Dührsen U (2004). Anti-CD20 monoclonal antibody therapy in relapsed MALT lymphoma of the conjunctiva. Eur J Haematol.

[56] Chanudet E, Zhou Y, Bacon CM, Wotherspoon AC, Müller-Hermelink HK, Adam P (2006). Chlamydia psittaci is variably associated with ocular adnexal MALT lymphoma in different geographical regions. J Pathol.

[57] Ferreri AJ, Ponzoni M, Guidoboni M, De Conciliis C, Resti AG, Mazzi B (2005). Regression of ocular adnexal lymphoma after Chlamydia psittaci-eradicating antibiotic therapy. J Clin Oncol.

[58] Tananuvat N, Lertprasertsuk N, Mahanupap P, Noppanakeepong P (2008). Role of impression cytology in diagnosis of ocular surface neoplasia. Cornea.

[59] Nolan GR, Hirst LW, Bancroft BJ (2001). The cytomorphology of ocular surface squamous neoplasia by using impression cytology. Cancer (Cancer Cytopathol).

[60] Keijser S, Missotten GS, De Wolff-Rouendaal D, Verbeke SL, Van Luijk CM, Veselic-Charvat M (2007). Impression cytology of melanocytic conjunctival tumours using the Biopore membrane. Eur J Ophthalmol.

[61] Paridaens AD, McCartney AC, Curling OM, Lyons CJ, Hungerford JL (1992). Impression cytology of conjunctival melanosis and melanoma. Br J Ophthalmol.

[62] Messmer EM, Mackert MJ, Zapp DM, Kampik A (2006). In vivo confocal microscopy of pigmented conjunctival tumors. Graefes Arch Clin Exp Ophthalmol.

[63] Shields JA, Shields CL, De Potter P (1997). Surgical management of conjunctival tumors: The 1994 Lynn B McMahan Lecture. Arch Ophthalmol.

[64] Shields JA, Shields CL, De Potter P (1998). Surgical management of circumscribed conjunctival melanomas. Ophthal Plast Reconstr Surg.

[65] Peksayar G, Soyturk MK, Demiryont M (1989). Long-term results of cryotherapy on malignant epithelial tumors of the conjunctiva. Am J Ophthalmol.

[66] Seregard S (1998). Conjunctival melanoma. Surv Ophthalmol.

[67] Shields CL (2000). Conjunctival melanoma: Risk factors for recurrence, exenteration, metastasis, and death in 150 consecutive patients. Trans Am Ophthalmol Soc.

[68] Lederman M, Wybar K, Busby E (1984). Malignant epibulbar melanoma: Natural history and treatment by radiotherapy. Br J Ophthalmol.

[69] Lommatzsch PK, Lommatzsch RE, Kirsch I, Fuhrmann P (1990). Therapeutic outcome of patients suffering from malignant melanomas of the conjunctiva. Br J Ophthalmol.

[70] Kenyon KR, Rapoza PA (1995). Limbal allograft transplantation for ocular surface disorders. Ophthalmology.

[71] Copeland RA, Char DH (1990). Limbal autograft reconstruction after conjunctival squamous cell carcinoma. Am J Ophthalmol.

[72] Puangsricharern V, Tseng SC (1995). Cytologic evidence of corneal diseases with limbal stem cell deficiency. Ophthalmology.

[73] Tseng SC, Sun TT, Brightbill FS (1999). Corneal surgery: Theory, technique, and tissue.

[74] Schwartz GS, Holland EJ (1998). Iatrogenic limbal stem cell deficiency. Cornea.

[75] Pires RT, Chokshi A, Tseng SC (2000). Amniotic membrane transplantation or limbal conjunctival autograft for limbal stem cell deficiency induced by 5- fluorouracil in glaucoma surgeries. Cornea.

[76] Tseng SC, Prabhasawat P, Barton K, Gray T, Meller D (1998). Amniotic membrane transplantation with or without limbal allografts for corneal surface reconstruction in patients with limbal stem cell deficiency. Arch Ophthalmol.

[77] Anderson DF, Ellies P, Pires RT, Tseng SC (2001). Amniotic membrane transplantation for partial limbal stem cell deficiency. Br J Ophthalmol.

[78] Prabhasawat P, Tseng SC (1997). Impression cytology study of epithelial phenotype of ocular surface reconstructed by preserved human amniotic membrane. Arch Ophthalmol.

[79] Meller D, Tseng SC (1999). Conjunctival epithelial cell differentiation on amniotic membrane. Invest Ophthalmol Vis Sci.

[80] Tsai RJ, Li LM, Chen JK (2000). Reconstruction of damaged corneas by transplantation of autologous limbal epithelial cells. N Engl J Med.

[81] Koizumi N, Inatomi T, Suzuki T, Sotozono C, Kinoshita S (2001). Cultivated corneal epithelial transplantation for ocular surface reconstruction in acute phase of Stevens-Johnson syndrome. Arch Ophthalmol.

[82] Anderson DF, Ellies P, Pires RT, Tseng SC (2001). Amniotic membrane transplantation for partial limbal stem cell deficiency: Long term outcomes. Br J Ophthalmol.

[83] Tabin G, Levin S, Snibson G, Loughnan M, Taylor H (1997). Late recurrences and the necessity for long-term follow-up in corneal and conjunctival intraepithelial neoplasia. Ophthalmology.

[84] Poothullil AM, Colby KA (2006). Topical medical therapies for ocular surface tumors. Semin Ophthalmol.

[85] Lee DA, Shapourifar-Tehrani S, Kitada S (1990). Effects of mithramycin, mitomycin, daunorubicin and bleomycin on human subconjunctival fibroblast attachment and proliferation. Invest Ophthalmol Vis Sci.

[86] Peters GF, Peckham M, Pinedo HM, Veronesi U (1995). Antimetabolites. Oxford textbook of oncology.

[87] Hirst LW (2007). Randomized controlled trial of topical mitomycin C for ocular surface squamous neoplasia: Early resolution. Ophthalmology.

[88] Salmon SE, Sartorelli AC, Katzung BG (1995). Cancer chemotherapy. Basic and clinical pharmacology.

[89] Frucht-Pery J, Sugar J, Baum J, Sutphin JE, Pe’er J, Savir H (1997). Mitomycin C treatment for conjunctival-corneal intraepithelial neoplasia: A multicenter experience. Ophthalmology.

[90] Wilson MW, Hungerford JL, George SM, Madreperla SA (1997). Topical mitomycin C for the treatment of conjunctival and corneal epithelial dysplasia and neoplasia. Am J Ophthalmol.

[91] Rozenman Y, Frucht-Pery J (2000). Treatment of conjunctival intraepithelial neoplasia with topical drops of mitomycin C. Cornea.

[92] Wilson MW, Hungerford JL, George SM, Madreperla SA (1997). Topical mitomycin C for the treatment of conjunctival and corneal epithelial dysplasia and neoplasia. Am J Ophthalmol.

[93] Shields CL, Naseripour M, Shields JA (2002). Topical mitomycin C for extensive, recurrent conjunctival-corneal squamous cell carcinoma. Am J Ophthalmol.

[94] Tunc M, Erbilen E (2006). Topical cyclosporine: A combined with mitomycin C for conjunctival and corneal squamous cell carcinoma. Am J Ophthalmol.

[95] Demirci H, McCormick SA, Finger PT (2000). Topical mitomycin chemotherapy for conjunctival malignant melanoma and primary acquired melanosis with atypia: Clinical experience with histopathologic observations. Arch Ophthalmol.

[96] Kurli M, Finger PT (2005). Topical mitomycin chemotherapy for conjunctival malignant melanoma and primary acquired melanosis with atypia: 12 years’ experience. Graefes. Arch Clin Exp Ophthalmol.

[97] Schallenberg M, Niederdräing N, Steuhl KP, Meller D (2008). Topical Mitomycin C as a therapy of conjunctival tumours. Ophthalmologe.

[98] Ando H, Ido T, Kawai Y, Yamamoto T, Kitazawa Y (1992). Inhibition of corneal epithelial wound healing: A comparative study of mitomycin C and 5-fluorouracil. Ophthalmology.

[99] Khong JJ, Muecke J (2006). Complications of mitomycin C therapy in 100 eyes with ocular surface neoplasia. Br J Ophthalmol.

[100] Giaconi JA, Karp CL (2003). Current treatment options for conjunctival and corneal intraepithelial neoplasia. Ocul Surf.

[101] Sturges A, Butt AL, Lai JE, Chodosh J (2008). Topical interferon or surgical excision for the management of primary ocular surface squamous neoplasia. Ophthalmology.

[102] Majewski S, Szmurlo A, Marczak M, Jablonska S, Bollag W (1994). Synergistic effect of retinoids and interferon alpha on tumor-induced angiogenesis: Anti-angiogenic effect on HPV-harboring tumor-cell lines. Int J Cancer.

[103] Gangemi JD, Pirisi L, Angell M, Kreider JW (1994). HPV replication in experimental models: effects of interferon. Antiviral Res.

[104] Vann RR, Karp CL (1999). Perilesional and topical interferon alfa-2b for conjunctival and corneal neoplasia. Ophthalmology.

[105] Huerva V, Mateo AJ, Mangues I, Jurjo C (2006). Short-term mitomycin C followed by long-term interferon alpha2beta for conjunctiva-cornea intraepithelial neoplasia. Cornea.

[106] Hu FR, Wu MJ, Kuo SH (1998). Interferon treatment for corneolimbal squamous dysplasia. Am J Ophthalmol.

[107] Karp CL, Moore JK, Rosa RH (2001). Treatment of conjunctival and corneal intraepithelial neoplasia with topical interferon alpha-2b. Ophthalmology.

[108] Kobayashi A, Yoshita T, Uchiyama K, Shirao Y, Kitagawa K, Fujisawa A (2002). Successful management of conjunctival intraepithelial neoplasia by interferon alpha-2b. Jpn J Ophthalmol.

[109] Boehm MD, Huang AJ (2004). Treatment of recurrent corneal and conjunctival intraepithelial neoplasia with topical interferon alfa 2b. Ophthalmology.

[110] Holcombe DJ, Lee GA (2006). Topical interferon alfa-2b for the treatment of recalcitrant ocular surface squamous neoplasia. Am J Ophthalmol.

[111] Schechter BA, Schrier A, Nagler RS, Smith EF, Velasquez GE (2002). Regression of presumed primary conjunctival and corneal intraepithelial neoplasia with topical interferon alpha-2b. Cornea.

[112] Smith M, Trousdale MD, Rao NA, Robin JB (1989). Lack of toxicity of a topical recombinant interferon alpha. Cornea.

[113] Finger PT, Sedeek RW, Chin KJ (2008). Topical interferon alfa in the treatment of conjunctival melanoma and primary acquired melanosis complex. Am J Ophthalmol.

[114] Nemet AY, Martin P, Benger R, Kourt G, Sharma V, Ghabrial R (2007). Orbital exenteration: A 15-year study of 38 cases. Ophthal Plast Reconstr Surg.

